# Species-specific differences in NPC1 protein trafficking govern therapeutic response in Niemann-Pick type C disease

**DOI:** 10.1172/jci.insight.160308

**Published:** 2022-12-08

**Authors:** Mark L. Schultz, Kylie J. Schache, Ruth D. Azaria, Esmée Q. Kuiper, Steven Erwood, Evgueni A. Ivakine, Nicole Y. Farhat, Forbes D. Porter, Koralege C. Pathmasiri, Stephanie M. Cologna, Michael D. Uhler, Andrew P. Lieberman

**Affiliations:** 1 Department of Pathology, University of Michigan Medical School, Ann Arbor, Michigan, USA.; 2 Program in Genetics and Genome Biology, The Hospital for Sick Children Research Institute, Toronto, Ontario, Canada.; 3 Department of Molecular Genetics and; 4 Department of Physiology, University of Toronto, Toronto, Ontario, Canada.; 5 Division of Translational Medicine, Eunice Kennedy Shriver National Institute of Child Health and Human Development, NIH, Department of Health and Human Services, Bethesda, Maryland, USA.; 6 Department of Chemistry, University of Illinois, Chicago, Illinois, USA.; 7 Michigan Neuroscience Institute and; 8 Department of Biological Chemistry, University of Michigan Medical School, Ann Arbor, Michigan, USA.

**Keywords:** Neuroscience, Therapeutics, Lysosomes, Protein misfolding, Protein traffic

## Abstract

The folding and trafficking of transmembrane glycoproteins are essential for cellular homeostasis and are compromised in many diseases. In Niemann-Pick type C disease, a lysosomal disorder characterized by impaired intracellular cholesterol trafficking, the transmembrane glycoprotein NPC1 misfolds due to disease-causing missense mutations. While mutant NPC1 has emerged as a robust target for proteostasis modulators, drug development efforts have been unsuccessful in mouse models. Here, we demonstrated unexpected differences in trafficking through the medial Golgi between mouse and human I1061T-NPC1, a common disease-causing mutant. We established that these distinctions are governed by differences in the NPC1 protein sequence rather than by variations in the endoplasmic reticulum–folding environment. Moreover, we demonstrated direct effects of mutant protein trafficking on the response to small molecules that modulate the endoplasmic reticulum–folding environment by affecting Ca^++^ concentration. Finally, we developed a panel of isogenic human NPC1 iNeurons expressing WT, I1061T-, and R934L-NPC1 and demonstrated their utility in testing these candidate therapeutics. Our findings identify important rules governing mutant NPC1’s response to proteostatic modulators and highlight the importance of species- and mutation-specific responses for therapy development.

## Introduction

Transmembrane glycoproteins are synthesized and folded in the endoplasmic reticulum (ER) by a complex, iterative process. In the ER lumen, cotranslational addition of glycans initiates glycoprotein folding (1). Chaperones then bind, modify, and release these glycoproteins to guide proper folding and ER export (1). Misfolded glycoproteins are recognized and degraded by ER quality control (ERQC) mechanisms, such as ER-associated degradation and ER-targeted macroautophagy. These processes are particularly important to human health, as mutations in many disease-associated genes lead to misfolded proteins that are subjected to ERQC (1, 2).

One such disease-associated gene is *NPC1*, which encodes a transmembrane glycoprotein that localizes to late endosomes/lysosomes (LE/Lys). Loss-of-function mutations in *NPC1* cause Niemann-Pick type C disease, an autosomal recessive lysosomal disorder characterized by the accumulation of unesterified cholesterol in the endolysosomal compartment (3). Niemann-Pick type C disease is a devastating illness that includes neonatal liver disease, followed by a gradually worsening neurological course, with loss of motor skills, cognitive decline, seizures, and, most often, death by early adolescence (4). The vast majority of Niemann-Pick C cases (~95%) are caused by mutations in the *NPC1* gene (5), with the remaining fraction (~5%) caused by mutations in *NPC2* (6). Although this is a rare disorder (~1:150,000 to 1:50,000) (4, 7), studies of Niemann-Pick C disease have provided key insights into mechanisms controlling intracellular cholesterol trafficking. Within the LE/Lys, soluble NPC2 binds cholesterol and transfers it to NPC1, a multipass transmembrane protein that facilitates cholesterol efflux from the endolysosomal compartment and subsequent redistribution (8–11). Consequences of disrupted intracellular cholesterol trafficking are diverse and include impairments of autophagy (12–19), lysosomal membrane permeabilization (20–22), mitochondrial abnormalities (17, 23), altered mTOR activity (21, 24), and ER calcium dysregulation (25). In aggregate, these effects result in neuron loss (26–28) and myelination defects (29) in the CNS.

Missense mutations in *NPC1* are the most common cause of Niemann-Pick C disease, with more than 250 disease-causing sequence variants identified throughout the gene. One mutation, an isoleucine-to-threonine substitution at position 1061 (I1061T), is particularly prevalent, occurring in approximately 20% of patients of Western European ancestry. Human I1061T-NPC1 misfolds in the ER, then is triaged by quality control machinery components, including the molecular chaperone calnexin (2). Through these interactions, I1061T-NPC1 is ubiquitylated and degraded by the proteasome through MARCH6-dependent ER-associated degradation (30). Concurrently, I1061T-NPC1 is also subject to degradation through FAM134B-dependent ER-targeted macroautophagy (30). Importantly, bypassing these ERQC pathways enables I1061T-NPC1 trafficking to the LE/Lys where it is still functional. This can be accomplished in vitro by transient overexpression of I1061T-NPC1 or calnexin and by treatment with small molecules that modulate the protein folding environment, such as HDAC inhibitors or ryanodine receptor (RyR) antagonists (31–33).

To test proteostatic therapies in vivo, gene targeting was previously used to generate mice with the I1061T mutation (ATA to ACA) inserted into the mouse *Npc1* gene. I1061T-NPC1 mice recapitulate disease phenotypes, including intracellular lipid accumulation, reduced body weight, impaired motor function, age-dependent Purkinje cell degeneration, and premature death (34). Notably, in these mice, several small molecules that showed promise in human cell culture models failed to exhibit efficacy (35, 36). Similar challenges have occurred in the study of other misfolded glycoproteins, including CFTR (37), yet the underlying mechanisms remain poorly defined.

Here, we studied NPC1 with the goal of defining factors that affect mutant protein trafficking and degradation to provide insights for therapy development. We demonstrate unexpected differences in trafficking through the medial Golgi between mouse and human I1061T-NPC1. We establish that these distinctions are governed by sequence differences between mouse and human NPC1 protein rather than by differences in the ER-folding environment. We then extended these observations to an additional NPC1 disease-causing mutation, R934L, in which we demonstrate direct mutation-specific effects of protein trafficking on the response to small molecules that target ER Ca^++^ release channels. Finally, we developed a panel of human isogenic induced pluripotent stem cells (iPSCs) and induced neurons (iNeurons) and demonstrated their utility in testing potential therapeutics for Niemann-Pick C disease. Our findings provide insight into the trafficking and degradation of glycoproteins, such as those mutated in lysosomal diseases, where proteostatic regulators are under active study for the treatment of a wide array of human disorders.

## Results

### Species-specific trafficking differences in I1061T-NPC1.

**Figure 1:**
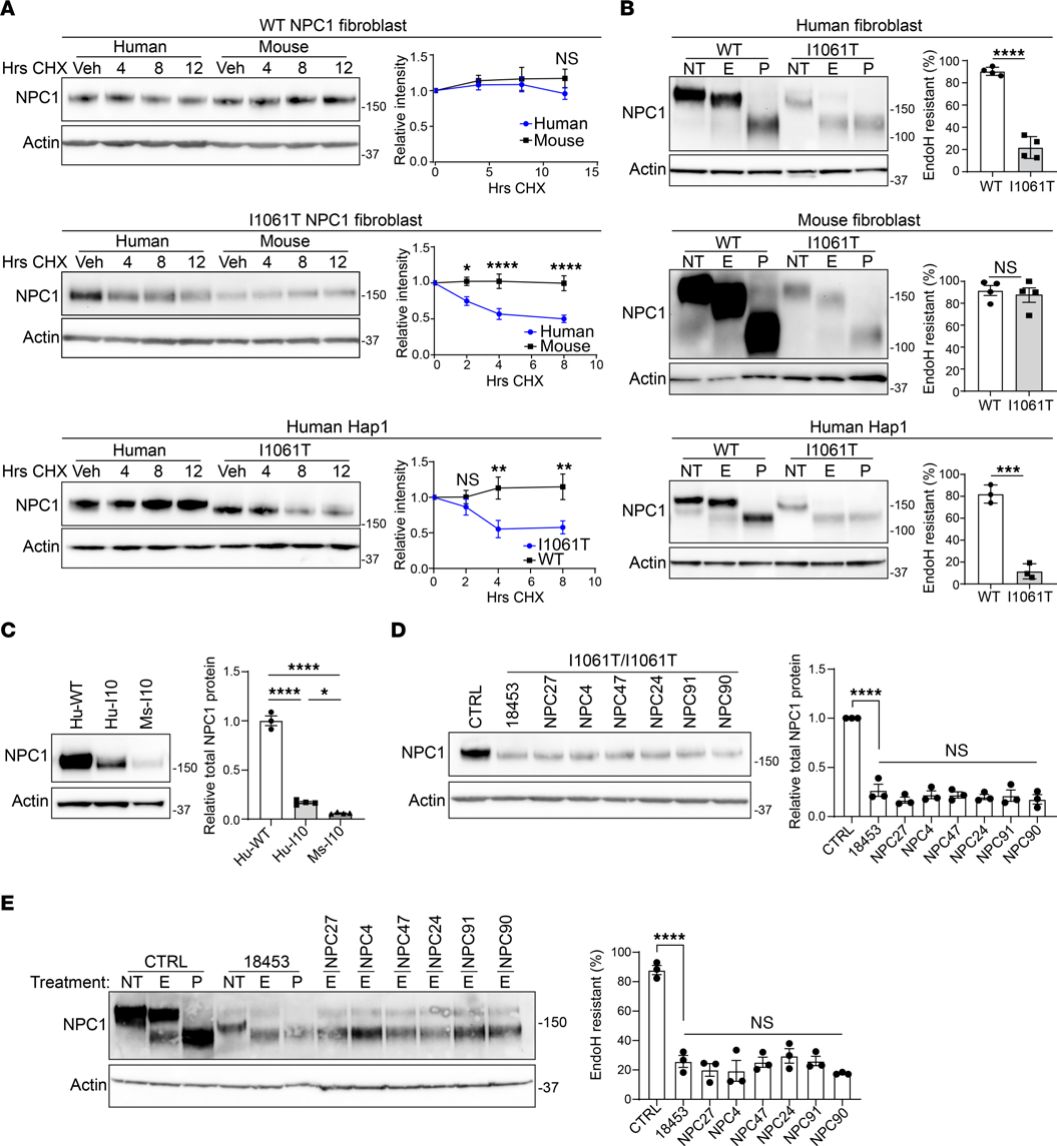
I1061T-NPC1 undergoes species-specific trafficking. (**A**) Human fibroblasts, mouse fibroblasts, and human Hap1 cells were treated with cycloheximide for the indicated times, and WT- or I1061T-NPC1 half-life was analyzed by Western blot and quantified. (**B**) Cell lysates from WT- or I1061T-NPC1 human fibroblasts, mouse fibroblasts, and human Hap1 cells were incubated with no treatment (NT) or digested with EndoH (E) or PNGase F (P) and then subjected to Western blot and quantified at right. (**C**) Lysates from human WT-NPC1 (Hu-WT), human I1061T/I1061T-NPC1 (Hu-I10), and mouse I1061T/I1061T-NPC1 (Ms-I10) fibroblasts were analyzed for total NPC1 by Western blot and quantified. (**D** and **E**) Lysates from a control cell line (CTRL; Coriell, GM08399) or a panel of I1061T/I1061T patient fibroblasts (Coriell, GM18453; NPC27, NPC4, NPC47, NPC24, NPC91, and NPC90) were analyzed for (**D**) total NPC1 protein or (**E**) incubated with NT or E and quantified. Data are shown as the mean ± SEM from (**A**) 6 to 8 or (**C–E**) 3 to 4 independent experiments. *P ≤ 0.05, **P ≤ 0.01, ***P ≤ 0.001, ****P ≤ 0.0001 by (**A**) 2-way ANOVA with Bonferroni’s multiple comparison test, (**B**) *t* test, or (**C–E**) 1-way ANOVA with Tukey’s post hoc test. (**A**) F = 0.6, 9.0, 6.0; df = 3. (**B**) *t* = 13.1, 0.5, 11.3; df = 6, 6, 4. (**C–E**) F = 420, 46.3, 30.5; df = 2, 7, 7. See complete unedited blots in the supplemental material.

While several strategies improve human I1061T-NPC1 trafficking and function in vitro (2), these have been unsuccessful when tested in the I1061T-NPC1 knockin mouse (35, 36). Here, we sought to investigate whether differences between mouse and human I1061T-NPC1 protein trafficking contribute to this discrepancy. Because NPC1 in its native conformation traffics to LE/Lys and is stable (31), we determined NPC1 half-life using a cycloheximide chase assay in primary human and mouse fibroblasts or human haploid (Hap1) cells (38). Both human and mouse WT-NPC1 had a half-life greater than 12 hours, consistent with a native, trafficked form (Figure 1A). In contrast, human I1061T-NPC1 in primary fibroblasts or Hap1 cells had a half-life of approximately 5–7 hours (Figure 1A), confirming previously reported measurements and indicating degradation from the ER (31, 34). Surprisingly, mouse I1061T-NPC1 (34) had a half-life similar to that of WT-NPC1 (Figure 1A), prompting us to examine trafficking through the medial Golgi and function in the LE/Lys of mouse and human I1061T-NPC1.

The degree of trafficking through the medial Golgi was assessed biochemically by taking advantage of the glycans that are added to NPC1 during folding and maturation. During synthesis in the ER, immature N*-*linked high mannose glycans are added to NPC1 (31). These glycans are sensitive to cleavage by the enzyme endoglycosidase H (EndoH), which is detected as enhanced migration on SDS-PAGE. As NPC1 traffics through the medial Golgi apparatus, these glycans are matured by glycan-modifying enzymes and become EndoH resistant. Thus, sensitivity to EndoH cleavage reflects NPC1 trafficking status relative to the Golgi. Due to misfolding and degradation from the ER, human I1061T-NPC1 is sensitive to EndoH cleavage (30, 31).

To determine whether the knockin mouse I1061T-NPC1 mimics human I1061T-NPC1 trafficking defects, we compared mouse and human I1061T-NPC1 EndoH sensitivity using primary fibroblasts. As a positive control, lysates were also treated with peptide-N-glycosidase F (PNGase F), an enzyme that cleaves all N-linked glycans (31). Human and mouse WT-NPC1 were resistant to EndoH (Figure 1B), indicating proper trafficking through the Golgi. Reflective of ER degradation, human I1061T-NPC1 was EndoH sensitive in both primary fibroblasts and previously described human Hap1 cells (Figure 1B) (38). Total human and mouse I1061T-NPC1 levels were reduced compared with those of WT-NPC1 (Figure 1C and Supplemental Figure 1, A and B; supplemental material available online with this article; https://doi.org/10.1172/jci.insight.160308DS1); however, the mutant mouse protein was, unexpectedly, EndoH resistant (Figure 1B). We confirmed species-specific EndoH sensitivity differences in 6 additional I1061T/I1061T primary patient fibroblast lines (Figure 1, D and E), indicating that species-specific EndoH differences were not due to genetic modifiers unique to one specific line of patient fibroblasts. Our findings reveal that remaining mouse I1061T-NPC1 protein traffics through the Golgi, demonstrating surprising differences between the human and mouse mutant proteins.

**Figure 2:**
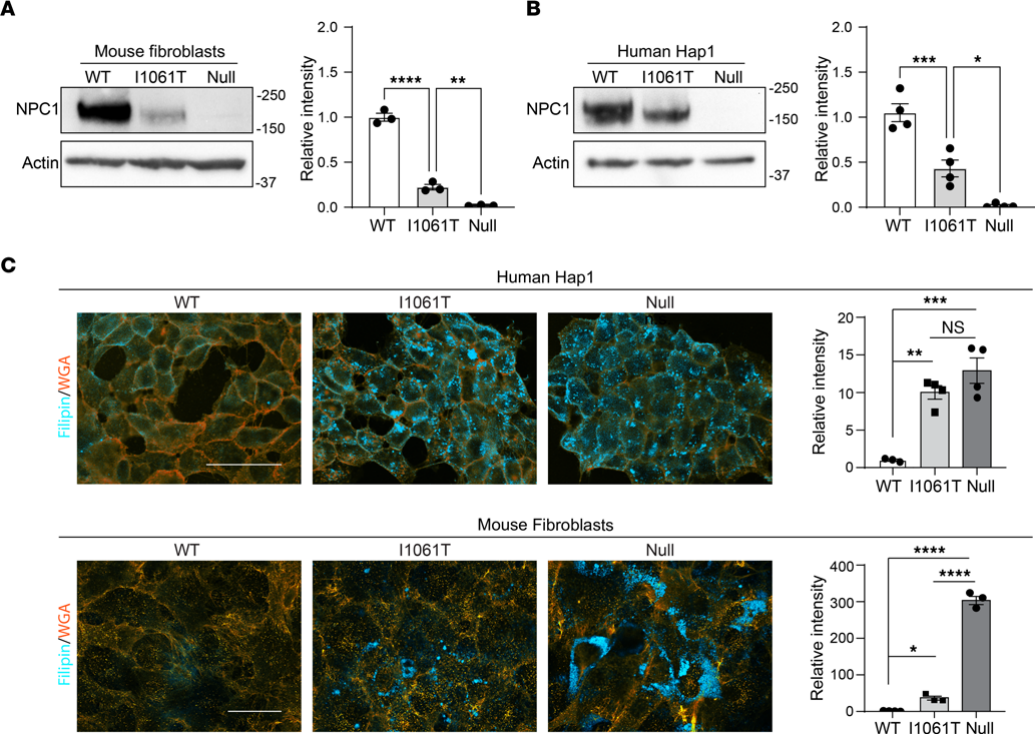
Mouse I1061T-NPC1 retains function. (**A** and **B**) Lysates from primary mouse fibroblasts and human Hap1 cells with WT-NPC1, I1061T-NPC1, or Null-NPC1 were analyzed by Western blot for total NPC1 and quantified. (**C**) Cholesterol storage was measured by filipin (cyan) staining in WT-NPC1, I1061T-NPC1, and NPC1-null human Hap1 cells or mouse fibroblasts. WGA (orange) was used to label plasma membranes. Scale bar: 40 μm. Filipin intensity was normalized relative to WT and quantified. Data are shown as the mean ± SEM from 3 to 4 independent experiments. *P ≤ 0.05, **P ≤ 0.01, ***P ≤ 0.001, ****P ≤ 0.0001 by ANOVA with Tukey’s post hoc. (**A** and **B**) F = 282.0, 43.5, 282.0; df = 2. (**C**) F = 23.4; 666.0; df = 2.

Because human I1061T-NPC1 is still functional if it traffics to the LE/Lys (31, 32), we sought to determine if mouse I1061T-NPC1 has a functional effect on Niemann-Pick C cellular pathology. Utilizing the knockin mouse I1061T-NPC1 allele *loxP* sites (34), we transiently expressed GFP-tagged Cre recombinase and selected cells to establish a mouse fibroblast line deficient in NPC1 (Figure 2A). This allowed us to compare cholesterol accumulation in isogenic WT-NPC1, I1061T-NPC1, and null-NPC1 mouse fibroblasts. Similarly, HAP1 cells, having a near haploid genome, provide a robust model system for genetic manipulation and enabled the prior generation of isogenic WT-NPC1, I1061T-NPC1, and null-NPC1 human cells (38) (Figure 2B). We assessed the accumulation of unesterified cholesterol in these mouse and human cells using the fluorescent dye, filipin. Filipin staining of human I1061T-NPC1 and null-NPC1 Hap1 cells was similar and significantly greater than that in WT-NPC1 Hap1 cells, indicating little functional I1061T-NPC1 trafficked to the lysosome (Figure 2C, top). In contrast, mouse I1061T-NPC1 cells had significantly less filipin staining than mouse null-NPC1 cells (Figure 2C, bottom). Consequently, we conclude that residual mouse I1061T-NPC1 functions at the LE/Lys to reduce cholesterol storage. However, due to low total protein levels (Figure 2A), mouse I1061T-NPC1 cells still accumulate significantly more cholesterol than their WT counterparts (Figure 2C).

### Species-specific differences in NPC1 protein sequence affect protein trafficking.

**Figure 3:**
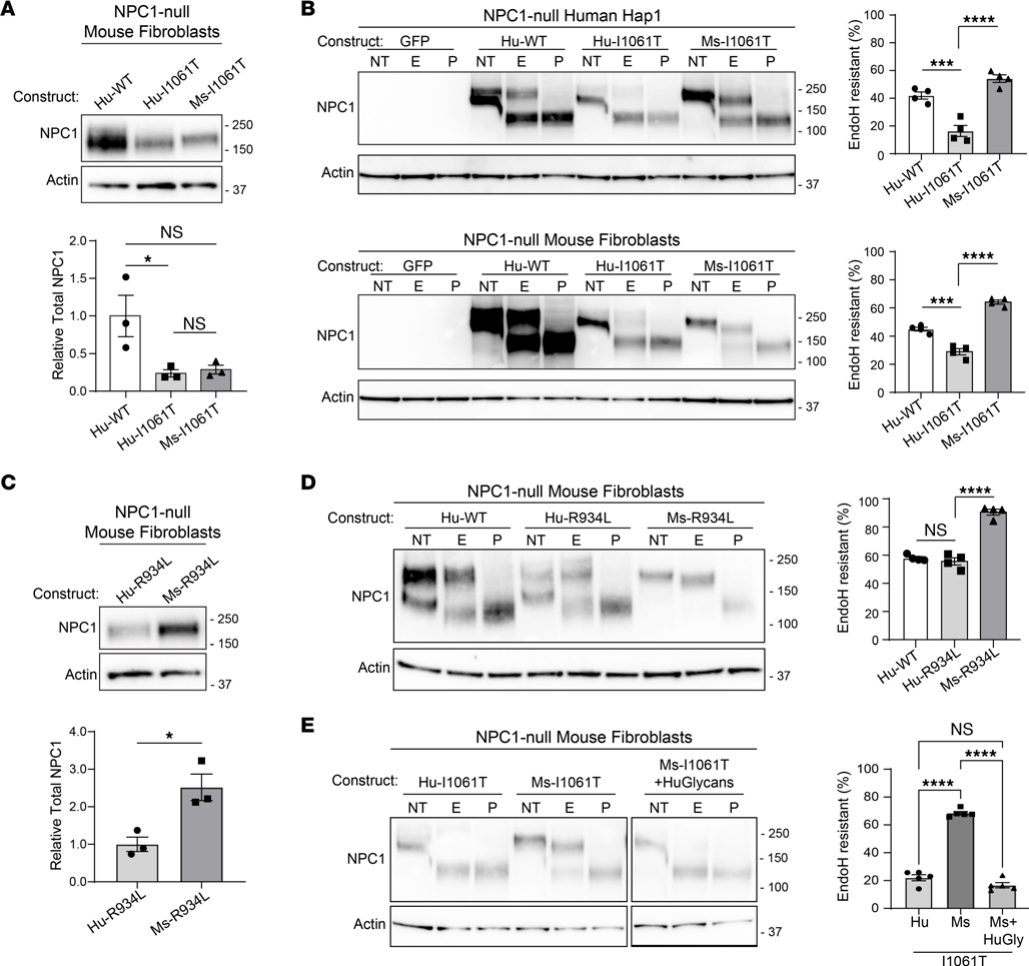
NPC1 protein sequence drives differential trafficking. (**A** and **C**) NPC1-deficient mouse fibroblasts were electroporated with (**A**) human WT, human I1061T, and mouse I1061T and (**C**) human R934L and mouse R934L, and total NPC1 was analyzed by Western blot and quantified. (**B**) Mouse fibroblasts and human Hap1 cells deficient in *NPC1* were electroporated with GFP, human WT-NPC1, human I1061T-NPC1, or mouse I1061T-NPC1 plasmids. Lysates were incubated with no treatment (NT) or digested with EndoH (E) or PNGase F (P) and then subjected to Western blot and quantified. (**D**) NPC1-deficient mouse fibroblasts were electroporated with human WT-NPC1, human R934L-NPC1, or mouse R934L-NPC1 plasmids, and lysates were treated as in **B** and quantified. (**E**) Hu-I1061T, Mu-I1061T, or Mu-I1061T containing the human glycosylation sites (Ms-I1061T+HuGlycans) were overexpressed and digested with E or P and quantified. Data are shown as the mean ± SEM from indicated number of independent experiments. *P ≤ 0.05, ***P ≤ 0.001, ****P ≤ 0.0001 by (**A**, **B**, **D**, and **E**) ANOVA with Tukey’s post hoc test or (**C**) *t* test. (**A** and **C**) *n* = 3, F = 6.7, *t* = 3.8, df = 2,4. (**B**) Hap1, Mef; *n* = 4,4; F = 37.8, 104.5; df = 2. (**D**) *n* = 4; F = 95.5; df = 2, (**E**) *n* = 5, F = 248.2, df = 2. See complete unedited blots in the supplemental material.

We hypothesized that species-specific differences in I1061T-NPC1 trafficking could be caused by differences in either the ER-folding environment or the NPC1 protein sequence. To distinguish between these possibilities, we transiently overexpressed identical vector backbones containing human WT-NPC1, human I1061T-NPC1, or mouse I1061T-NPC1 into null-NPC1 human or mouse cells and compared trafficking through the medial Golgi. In these systems, the total amount of human and mouse I1061T-NPC1 was similar (Figure 3A). Importantly, irrespective of each species’ folding environment, mouse I1061T was significantly more EndoH resistant (i.e., trafficked) than human I1061T (Figure 3B). To further support that species-specific trafficking was folding-environment independent, we tested the more efficiently trafficked R934L-NPC1 mutation (38). Likely reflecting reduced degradation, the total amount of overexpressed mouse R934L was greater than that of human R934L (Figure 3C). Expression of human R934L-NPC1 produced both EndoH-sensitive and -resistant forms (Figure 3D). In contrast, mouse R934L was nearly all EndoH resistant (Figure 3D), indicating again that the mouse protein trafficked more efficiently than the human protein. Overall, these data indicate that, despite 86% identity and 93% similarity of human and mouse NPC1 proteins, sequence differences are responsible for species-specific trafficking.

Because glycans contribute to the regulation of native protein folding and trafficking, we wondered if species-specific differences in NPC1 glycosylation could underlie trafficking differences between mouse and human I1061T-NPC1. N-linked glycans are added to the asparagine residue of the acceptor sequence N-X-S/T/C (39). Using this consensus sequence we employed prediction software (NetNGlyc, https://bio.tools/netnglyc; GlycoEP, https://bio.tools/glycoep) to identify likely sites of glycan addition (40, 41). In conjunction with a published human NPC1 crystal structure (11), these programs allowed us to identify 3 potential glycosylation sites (N452, N931, N1072) in the human protein that were absent in mouse NPC1. We inserted these sites by mutating the mouse protein sequence to the acceptor sequence present in human NPC1. Addition of these 3 glycan sites caused mouse I1061T-NPC1 trafficking to mimic human I1061T-NPC1 (Figure 3E), highlighting the importance of glycans in the regulation of human I1061T-NPC1 folding and trafficking.

### Species-specific response to RyR antagonists.

**Figure 4:**
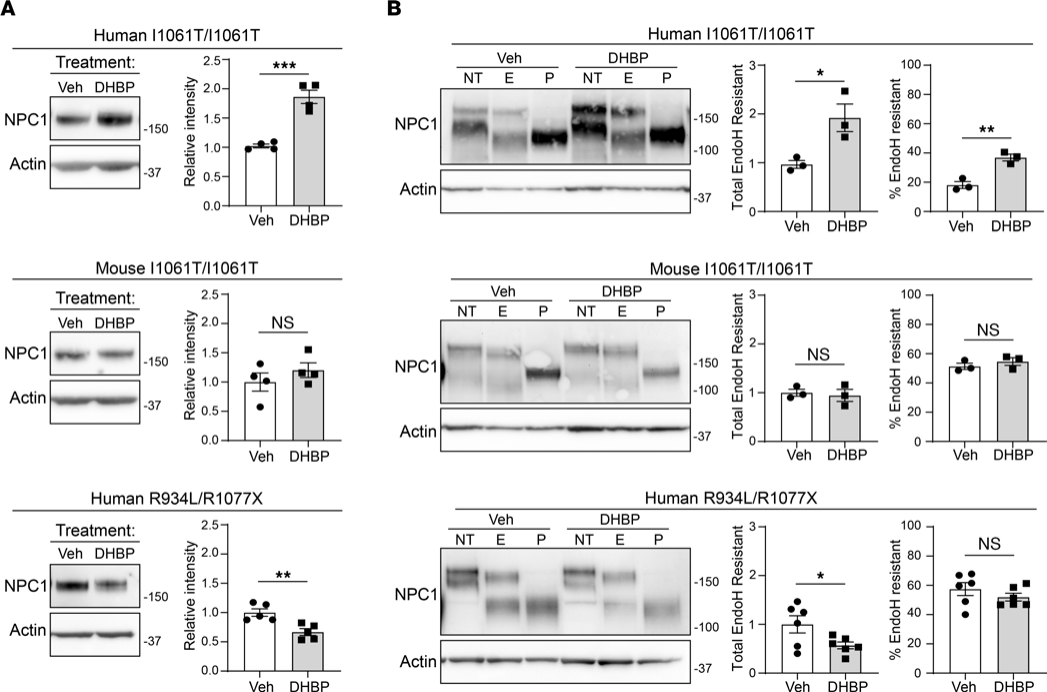
Species- and mutation-specific response to DHBP. (**A**) Primary human and mouse I1061T/I1061T or human R934L/R1077X fibroblasts were treated with 5 μM DHBP or vehicle for 5 days and analyzed by (**A**) Western blot for total NPC1 (quantified at right) or (**B**) incubated with no treatment (NT) or digested with EndoH (E) or PNGase F (P) to assess trafficking (quantified at right). Data are shown as the mean ± SEM from indicated number of independent experiments. ***P ≤ 0.001, ****P ≤ 0.0001 by *t* test. (**A**) Human I1061T, mouse I1061T, human R934L; *n* = 4, 4, 5; *t* = 7.1, 1.0, 3.8; df = 6, 6, 8; (**B**) Human I1061T, mouse I1061T, human R934L; *n* = 3, 3, 3, 3, 6, 6; *t* = 3.2, 5.7, 0.4, 1.0, 2.3, 1.1. df = 4, 4, 4, 4, 10, 10. See complete unedited blots in the supplemental material.

**Figure 5:**
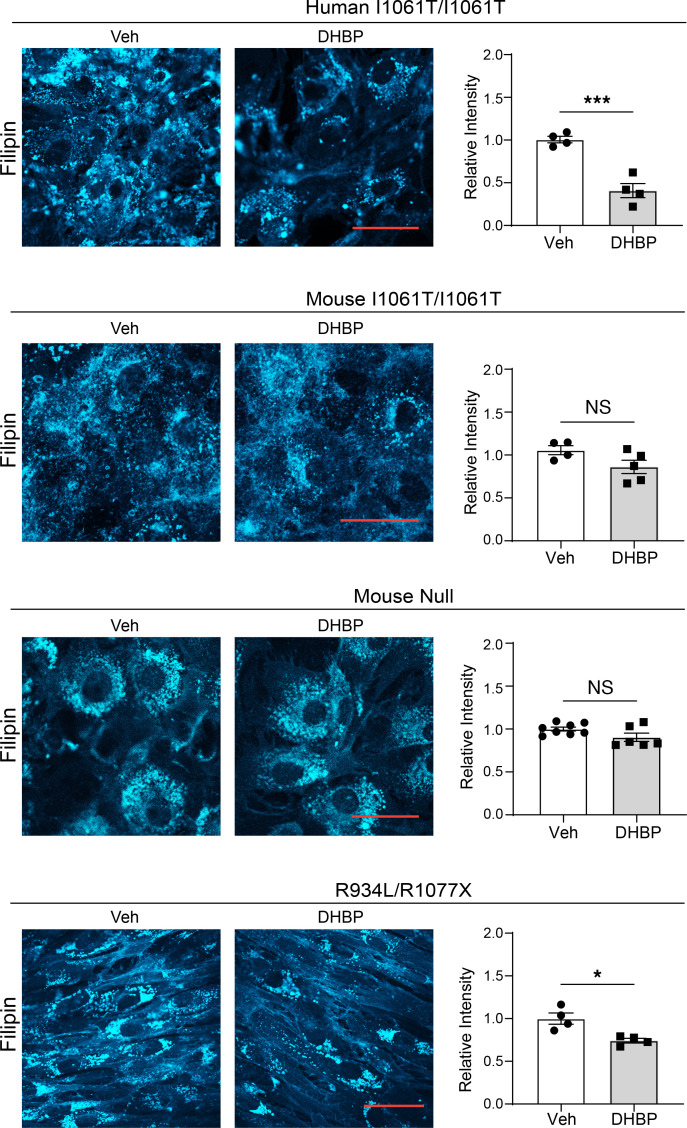
Mutation-specific response to DHBP. I1061T/I1061T or NPC1-null primary human and mouse fibroblasts or R934L/R1077X human fibroblasts were treated with vehicle or 5 μM DHBP for 5 days. Cells were fixed and unesterified cholesterol was labeled with filipin (cyan) and quantified. Scale bar: 50 μm. Data are shown as the mean ± SEM from indicated number of independent experiments. ***P ≤ 0.001, by *t* test. Human I1061T, mouse I1061T, mouse null, R934L; *n* = 4–8; *t* = 6.6, 2.0, 1.9, 3.6; df = 6, 7, 12, 6.

We hypothesized that the extent of ER retention would affect the response of mutant NPC1 proteins to certain potential therapeutics. In contrast to human I1061T-NPC1, enhanced LE/Lys trafficking of mouse I1061T-NPC1 suggested that small molecules that modify the ER-folding environment would be less effective on mouse I1061T-NPC1 or on human NPC1 mutants with greater trafficking through the medial Golgi, such as R934L. To test this, we treated primary human and mouse I1061T/I1061T fibroblasts with the RyR antagonist 1,1′-diheptyl-4,4′-bipyridium dibromide (DHBP) for 5 days. Consistent with previous data (32), DHBP increased total and EndoH-resistant human I1061T-NPC1 without increasing WT-NPC1 protein levels or NPC1 gene expression (Figure 4 and Supplemental Figure 2, A and B). However, DHBP did not increase either total or EndoH-resistant mouse I1061T-NPC1 (Figure 4). Similarly, human R934L-NPC1 did not respond to DHBP (Figure 4) when studied in patient fibroblasts expressing only the R934L allele (38). Consistent with its role as a RyR inhibitor, DHBP did not alter the total levels of several chaperones, including Hsp70, Hsp90, Hsp40, or Bip (Supplemental Figure 2, C–E). DHBP robustly reduced filipin staining in human I1061T-NPC1 by more than 50% but had only a slight effect on human R934L-NPC1 fibroblasts and no effect on mouse I1061T-NPC1 or mouse null-NPC1 fibroblasts (Figure 5). Our findings support the notion that NPC1 mutants with substantial impairments of trafficking out of the ER are most responsive to RyR inhibition.

### RyR antagonists rescue lipid storage in human iNeurons.

**Figure 6:**
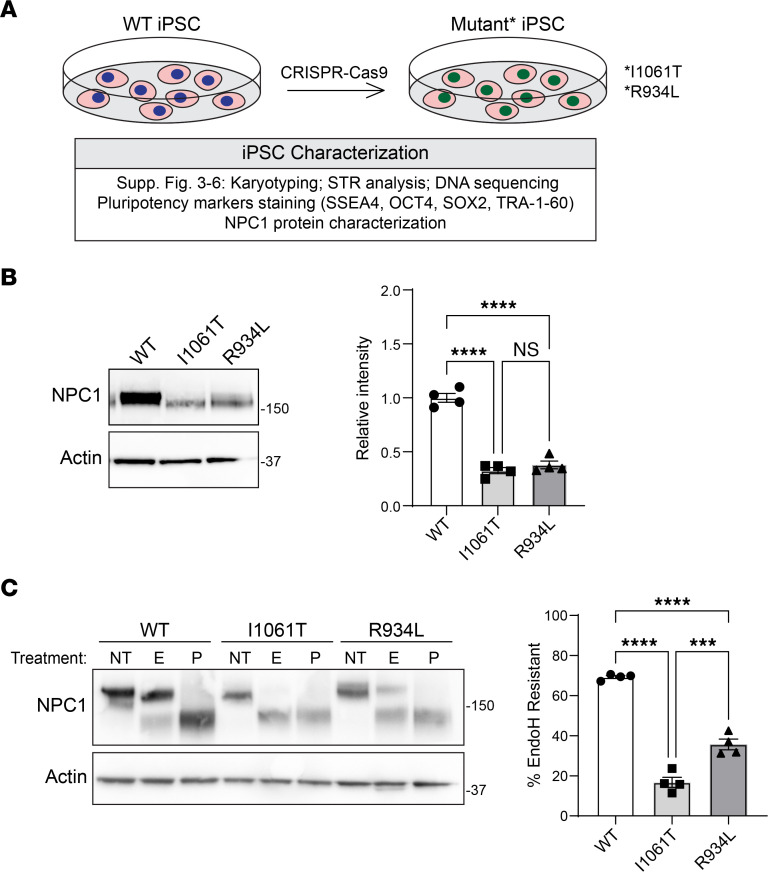
Creation of human iPSC NPC1 mutants. (**A**) Workflow describing the generation of human iPSC lines. (**B** and **C**) Lysates were collected from WT, I1061T, and R934L-NPC1 iPSCs and analyzed for (**B**) total NPC1 (quantified at right) or (**C**) incubated with no treatment (NT) or digested with EndoH (E) or PNGase F (P) (quantified at right). Data are shown as the mean ± SEM from 4 independent experiments. ***P ≤ 0.001, ****P ≤ 0.0001 by ANOVA with Tukey’s post hoc. (**B**) F = 111.6, df = 2. (**C**) F = 152.5; df = 2.

**Figure 7:**
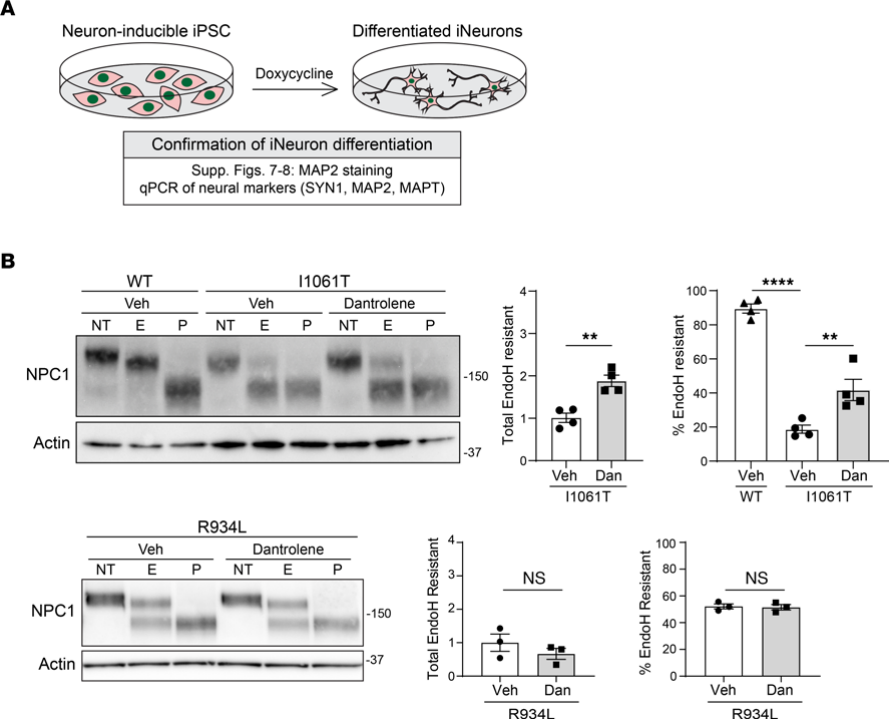
Mutation-specific response to dantrolene in iPSC-derived neurons. (**A**) Schematic depicting iNeuron generation and confirmation. (**B**) Isogenic WT, I1061T, and R934L iNeurons were treated with vehicle (Veh) or dantrolene (10 μM) for 5 days. Lysates were incubated with no treatment (NT) or digested with EndoH (E) or PNGase F (P) (quantified at right). Data are shown as the mean ± SEM from 3 to 4 independent experiments. **P ≤ 0.01, ****P ≤ 0.0001 by *t* test or ANOVA with Tukey’s post hoc. (**B**) I1061T, *t* = 5.3, F = 75.1, df = 6,9; R934L, *t* = 1.1, 0.2, df = 4.

We next sought to study NPC1 function and trafficking in human neurons, key target cells in Niemann-Pick C neuropathology (26–28). To accomplish this, we used the BJFF.6 iPSC line (42) and CRISPR/Cas9 gene editing to generate isogenic WT, I1061T, and R934L-NPC1 iPSCs (Figure 6A). These cells were characterized by normal karyotypes and pluripotency (Supplemental Figures 3 and 4 and Supplemental Table 1). Two independent I1061T and R934L iPSC clones exhibited diminished total NPC1 protein levels and impaired trafficking through the Golgi, as reflected by increased EndoH sensitivity relative to WT-NPC1 (Figure 6, B and C, and Supplemental Figure 5). These findings indicated that NPC1 trafficking in iPSCs recapitulates what is observed in human fibroblasts. Next, we used PiggyBac transposase to insert a doxycycline inducible neurogenin-2 cassette (43) in 2 independent clones derived from each NPC1 iPSC line. This system enabled rapid and efficient differentiation of isogenic iPSCs into iNeurons-expressing neuronal genes and displaying neuronal morphology over a period of 5 days (Figure 7A and Supplemental Figures 6 and 7).

iNeurons were treated for 5 days with vehicle or a nontoxic dose (Supplemental Figure 8A) of dantrolene, an FDA-approved RyR antagonist. Like patient fibroblasts, dantrolene significantly rescued I1061T-NPC1 trafficking in neurons, increasing both the total amount of EndoH-resistant protein and the percentage of EndoH-resistant I1061T-NPC1 (Figure 7B and Supplemental Figure 8C) without altering NPC1 gene expression (Supplemental Figure 8B). In contrast, dantrolene had no effect on R934L-NPC1 (Figure 7B and Supplemental Figure 8C), supporting the conclusion that mutant proteins more substantially retained within the ER are more responsive to RyR inhibitors.

**Figure 8:**
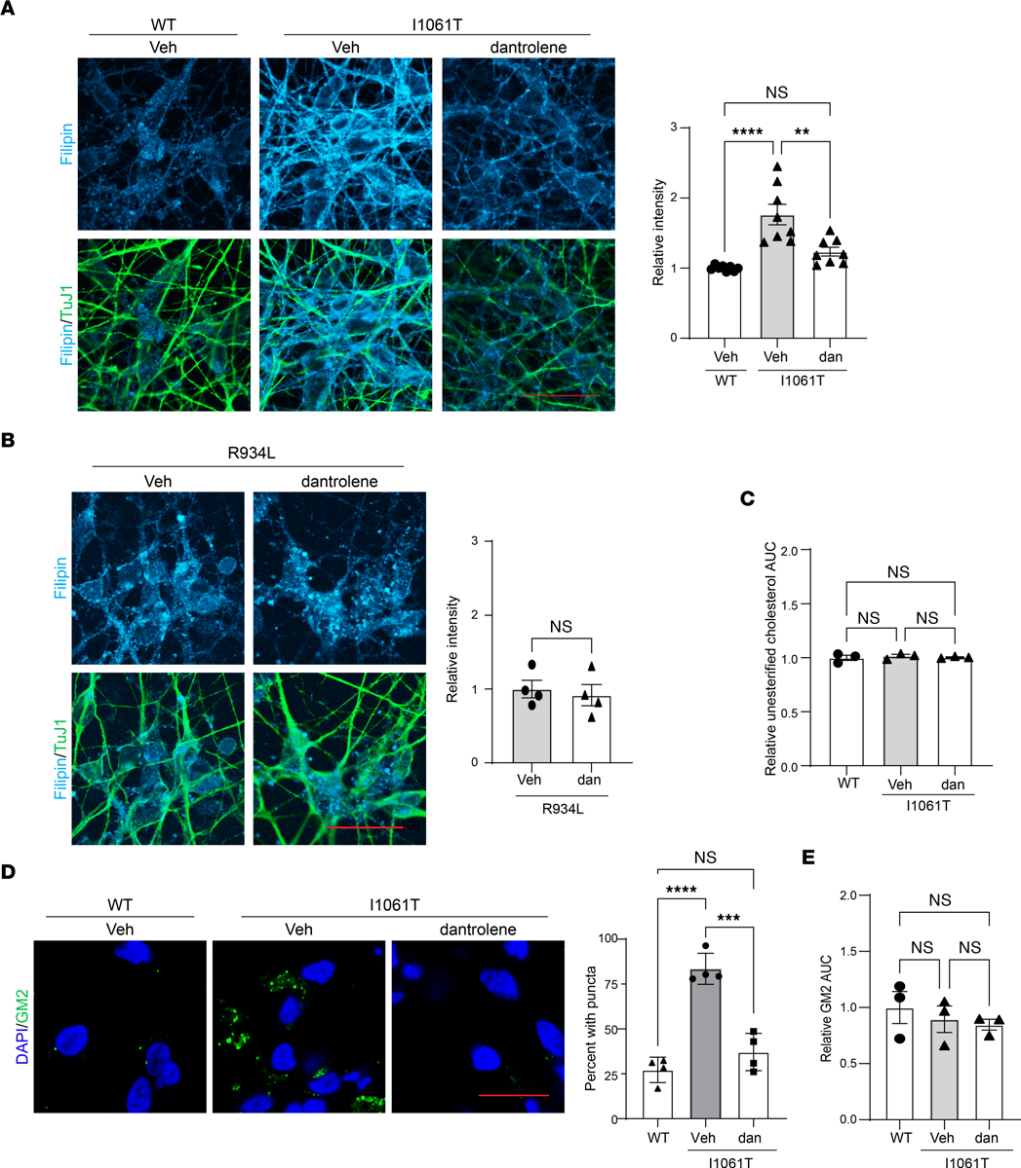
Dantrolene corrects lipid imbalances in I1061T-NPC1–induced neurons. Isogenic WT, I1061T, or R934L iNeurons were treated with vehicle (Veh) or dantrolene (dan; 10 μM) for 5 days. (**A** and **B**) Cells were stained for TuJ1 (green), and unesterified cholesterol was labeled with filipin (cyan). Filipin intensity/TuJ1 quantified at right. (**C**) LC-MS analysis of relative unesterified cholesterol (area under the curve [AUC]). (**D**) Cells were stained for GM2 (green). Nuclei were stained with DAPI (blue). Percentage of cells with GM2 puncta are quantified. (**E**) LC-MS analysis of relative GM2 (34:1) (AUC). Scale bars: 30 μm. Data are shown as the mean ± SEM from (**A**) 8, (**B** and **D**) 4, or (**C** and **E**) 3 independent experiments. **P ≤ 0.01, ***P ≤ 0.001, ****P ≤ 0.0001 by ANOVA with Tukey’s post hoc or *t* test. (**A**) F = 17.9, DF = 2; (**B**) *t* = 0.4 DF = 6; (**C**) F = 0.3, DF = 2; (**D**) F = 46.47, DF = 2; (**E**) F = 0.5, DF = 2.

Consistent with its effect on NPC1 trafficking, dantrolene reduced filipin staining of I1061T-NPC1 but not R934L-NPC1 iNeurons (Figure 8, A and B), indicative of functional rescue of the ER-retained mutant. Interestingly, liquid chromatography–mass spectrometry (LC-MS) analysis of WT-NPC1, I1061T-NPC1, and dantrolene-treated I1061T-NPC1 iNeurons revealed no significant differences in unesterified cholesterol content (Figure 8C), consistent with prior data supporting a predominant defect in intracellular cholesterol trafficking in mutant neurons (44). Similarly, GM2 ganglioside, a secondary lipid stored in NPC1-deficient neurons, significantly accumulated in I1061T-NPC1 iNeurons and was rescued by dantrolene treatment (Figure 8D). LC-MS detected no significant differences in total GM2 abundance in iNeurons (Figure 8E), indicating that, like cholesterol, GM2 accumulation in these cells largely reflected defects in intracellular trafficking. Together, our data indicate that RyR inhibition rescues trafficking and function of I1061T-NPC1, but not R934L-NPC1, in human iNeurons, demonstrating mutation-specific response to a potential therapeutic in a critical target cell in disease.

## Discussion

Efforts to identify therapeutics for Niemann-Pick C include studies to develop proteostatic modulators that promote the proper folding and trafficking of mutant NPC1. This work has been bolstered by the discovery that human I1061T-NPC1 is functional if it escapes ERQC degradation and traffics to the LE/Lys (31). Furthermore, detailed categorization of the effects of missense mutations in NPC1 on protein folding and trafficking enabled the identification of 4 distinct classes of mutant human proteins (45). These include ones that are predominantly degraded from the ER, such as I1061T, and those that traffic to LE/Lys, albeit less efficiently than WT protein, such as R934L. Here, we studied these 2 NPC1 mutants as exemplars of their classes, with the goal of defining the rules that guide the response to small molecules that target aspects of the folding environment. The observations that we report have clear implications for Niemann-Pick C therapy development and will likely inform similar efforts underway to identify treatments for other lysosomal disorders.

Our investigations led to the unexpected discovery of marked differences in trafficking through the medial Golgi between mouse and human I1061T-NPC1. While both proteins misfold in the ER and are partially degraded, the remaining mouse protein efficiently traffics through the Golgi apparatus to LE/Lys where it functions in cholesterol efflux. Human I1061T-NPC1, in contrast, fails to advance from the ER and has no detectable function in LE/Lys. These distinctions are not due to inherent differences in the ER-folding environments between humans and mice, but instead are attributable to protein sequence differences. Specifically, our analysis suggests that species-specific differences in N-linked glycosylation are sufficient to account for the failure of human I1061T-NPC1 to advance from the ER. Whether alternative unique glycan sites also enable efficient trafficking of mouse I1061T-NPC1 is yet to be established. Nonetheless, our studies highlight fundamental, species-specific differences in NPC1 trafficking. Moreover, this functional assessment provides mechanistic insight into why the knockin I1061T-NPC1 mouse develops a phenotype that is milder than the null NPC1 mouse, including longer survival (34).

The significance of these differences in NPC1 trafficking is evident from studies of the response to RyR antagonists, inhibitors of ER Ca^++^ efflux channels. These small molecules have shown efficacy in prior studies of human fibroblasts from patients with Niemann-Pick C and Gaucher disease (32, 46). The ER-retained human I1061T protein responds to treatments with RyR antagonists by showing enhanced trafficking and function, while mouse I1061T-NPC1 is unresponsive. The effects of RyR antagonists are of particular interest, as recent analyses have shown that neurons deficient in NPC1 have significantly lower ER calcium stores (47), a finding that may indicate impaired function of calcium-dependent ER chaperones, such as calnexin. The observations made in studies of I1061T-NPC1 were corroborated by analysis of R934L-NPC1, a mutation that triggers only mild retention in the ER. Once again, the mouse mutant traffics much more efficiently than the human mutant. Notably, human R934L-NPC1 shows no significant change in trafficking in response to treatment with a RyR antagonist, supporting the conclusion that ER-retained mutants are most responsive to ER-targeted small molecules. We suggest that alternative strategies will be more effective against the class of mutants that includes R934L and traffics to LE/Lys with modest efficiency.

The findings reported here provide a compelling rationale for the need to study NPC1 trafficking and function in systems that faithfully model the human disease. While gene-targeted I1061T-NPC1 mice display many aspects of the Niemann-Pick C phenotype, the behavior of the mutant mouse protein indicates that this is not an appropriate model system for the study of certain therapeutic approaches. In contrast, the panel of isogenic iPSCs and iNeurons described here demonstrate a clear therapeutic response of I1061T-NPC1 to RyR antagonists. These cells will likely prove useful in the analysis of additional small molecules by providing a system to study the mutant human protein in a critical target cell of Niemann-Pick C neuropathology. Taken together, our findings identify important rules governing the response of mutant NPC1 to RyR antagonists and highlight the importance of species- and mutation-specific responses for therapy development.

## Methods

Further information can be found in Supplemental Methods.

### Antibodies

#### Primary antibodies.

The following primary antibodies were used: NPC1, 1:500, Abcam, catalog ab134113/clone EPR5209; Actin, 1:2,000, MilliporeSigma, catalog A5441/clone AC-15; TuJ1, 1:1500, Promega, catalog G712A/clone 5G8; and Map2, 1:150, MilliporeSigma, catalog MAB3418/clone AP20. Anti-GM2, 1:100 (20), was a gift from Konstantin Dobrenis (Albert Einstein College of Medicine, New York, New York, USA).

#### Secondary antibodies.

The following secondary antibodies were used (antibody, dilution, vendor): goat anti-mouse IgG (H+L)-HRP conjugate, 1:2,000, Bio-Rad, 170-6516; goat anti-rabbit IgG (H+L)-HRP conjugate, 1:2,000, Bio-Rad, 170-6515; and Alexa Fluor 488 goat anti-mouse IgG (H+L), 1:500, Invitrogen, A11029.

### Plasmids

All NPC1 plasmids were created by VectorBuilder using the VectorBuilder mammalian gene expression vector with an EF1A promoter, Kozak, and SV40 late pA. Mouse NPC1 (NM_008720.2) and human NPC1 (NM_000271.5) were used as the base sequences for adding mutations. GFP-Cre was obtained from Addgene, 11923, pBS598 EF1alpha-EGFPcre.

### Cell lines

The following cell lines were obtained from the National Institute of General Medical Sciences Human Genetic Cell Repository at the Coriell Institute for Medical Research: GM08399 (control) and GM18453 (I1061T/I1061T). All fibroblasts were grown in Advanced MEM (Gibco, 12492-013) plus 10% FBS (R&D Systems, S11150) plus PSG (Gibco, 10378016). WT, I1061T-NPC1, and null-NPC1 human Hap1 cells were previously described (38) and cultured in IMDM (Gibco, 12440061) plus 10% FBS.

#### Generation of primary mouse fibroblasts.

Using P2 mice, a 1–2 mm piece of tail was rinsed once in 70% ethanol and then twice in PBS containing 1X Antibiotic-Antimycotic (Gibco, 15240096). Next, tails were minced in 0.25 mL mixture of collagenase (Worthington Labs, LS004204, diluted in RPMI1640 plus L-glut, Gibco, 11875). After mincing, an additional 0.25 mL collagenase mixture was added, and the tissue was incubated for 30 minutes at 37°C. 6 mL MEF media (DMEM/F12 Gibco, 11330 + 10% FBS + PSG) was added, and tissues were incubated overnight at 37°C in 5% CO_2_. Cells were incubated for an additional 3 days to allow adherence and spread away from remaining tissue. Tissue pieces were removed using sterile instruments, and cells were split and cultured as described above.

#### Generation of NPC1-null fibroblasts.

Primary I1061T/I1061T mouse fibroblasts were electroporated with a plasmid containing GFP-Cre and FACS sorted to select GFP^+^ cells.

#### iPSCs.

NPC1 mutant iPSC lines were generated by the Genome Engineering and iPSC Center at Washington University in St. Louis (St. Louis, Missouri, USA). Using the previously characterized parental BJFF.6 iPSC line (42), gRNAs were designed to target close to the mutation site, and a single-strand DNA donor was designed to ensure that no recutting occurred after correction. Single-cell iPSC clones were screened for point mutations, and 2 independent clones were selected for iNeuron generation. iPSCs were cultured on Geltrex (Thermo Fisher, A1413302) in StemFlex Medium (Gibco, A33494-01) and split using Gentle Cell Dissociation Reagent (Stemcell, 100-0485).

#### Generation of isogenic induced neuronal cells.

iNeuron generation was based on previously described protocols (43). To increase genome integration, the mCherry, puromycin, and hNGN2 plasmids were moved into the PiggyBac system. Briefly, the parental WT control, and 2 independent clones of each *I1061T-NPC1* and *R934L-NPC1* iPSCs were plated on plates with coated Matrigel (Corning, 354277) in the presence of 1 μM Y-26732 (Cayman Chemical, 10005583) in StemFlex (Gibco, A3349401). On day 1, the media were changed to E8 (Stemcell, 05990) alone, and cells were transfected using 10 μL Lipofectamine Stem Transfection Reagent (Invitrogen, STEM00015) diluted in 125 μL Opti-MEM (Gibco, 31985062). 0.9 μg PBase, 0.7 μg mCherry, 0.7 μg hNGN2, and 0.2 μg Puromycin plasmids were mixed in 125 μL Opti-MEM. On day 2, the media were changed to StemFlex. On day 3, cells were lifted into a single-cell dilution series using Accutase (Stem Cell, 07922); cells were added into several culture plates with StemFlex and Y-27632. On days 4–5, cells were fed with StemFlex. Antibiotic selection was performed on day 6; StemFlex media were replaced with StemFlex containing 0.85 μg/mL puromycin (Invivogen, ant-pr-1). Selection was observed on day 7–8. On day 9–14, 10 mCherry^+^ colonies were selected, and neuron differentiation was characterized to identify the most efficient clones.

#### iNeuron differentiation.

On day –1, iPSCs were dissociated with gentle cell dissociation reagent (Stem Cell, 100-0485) and plated on Geltrex-coated plates (Thermo Fisher, A1413302) as a single-cell suspension with StemFlex media and Y-27632. On day 0, media were changed to StemFlex plus 0.5 μg/mL doxycycline (MilliporeSigma, D9891). On day 1, media were refreshed with StemFlex plus doxycycline. On day 2, cells were washed with 1× DPBS (Gibco, 14190-144), treated with accutase to create a single-cell suspension, and replated onto Matrigel and cultured with 3 N media (4 mL pen/strep, Gibco, 15140; 250 mL DMEM/F12, Gibco, 11320-033; Neurobasal Gibco, 21103-049; 125 μL 10 mg/mL insulin, MilliporeSigma, I9278; 2.5 mL Non Essential Amino Acids, Gibco, 11140-050; 2.5 mL N 2 supplement, Gibco, 17502-048; 5 mL B27 supplement, Gibco, 17504-044; 2 μL b-mercaptoethanol stock [12 M], MilliporeSigma, M7522; and 2.5 mL Glutamax Gibco, 35050-061 containing Y-27632 and doxycycline). Media were replaced with 3 N plus doxycycline on day 3. On day 4 and on, one-half of media were replaced daily with 3 N + doxycycline.

### Cycloheximide chase

Cells were treated with 60 μg/mL cycloheximide (MilliporeSigma, C7698) for the indicated times.

### Transfection

#### Electroporation of mouse null fibroblasts.

Primary fibroblasts were electroporated using the Neon (Thermo Fisher) transfection system. Briefly, cells were trypsinized, counted, and pelleted (400*g*, 5 minutes). The cell pellet was resuspended in buffer E2, TE, and plasmid and electroporated in 10 μL tips (1,700 volts, 20 width, 1 pulse).

#### Transfection of Hap1 cells.

Hap1 cells were transfected using Lipofectamine 3000 (Invitrogen), P3000, OPTI-MEM, and plasmid.

### DHBP/dantrolene treatment

Cells were treated with 5 μM DHBP (MilliporeSigma, 180858) or 10 μM dantrolene (MilliporeSigma, D9175) over the course of 5 days. iNeurons were treated starting on day 3 of differentiation, with half media changes each following day. Upon the completion of the respective treatments, media were removed, and cells were washed with PBS (Gibco, 10010-023) and collected.

### Cell collection and protein calculation

After aspirating cell media, PBS was added, and cells were removed with a cell scraper and centrifuged at 1,000*g* at 4°C for 5 minutes. The cell pellet was then resuspended in 100 μL RIPA (Teknova, R3792), cOmplete Mini protease inhibitor (Roche, 46264500), and 0.625 mg/mL N-ethylmaleimide (Acros Organics, 128-53-0) and sonicated until cells were completely dissociated. The resulting solution was then centrifuged at 3,000*g* for 5 minutes at 4°C, after which the supernatant, including suspended protein, was transferred. Protein concentrations were calculated using the DC-protein assay (Bio-Rad) and normalized.

### EndoH assay

The EndoH assay was performed as previously described (30) with slight modifications. Briefly, 20 μL reactions containing cell lysate, 2 μL glycoprotein denaturing buffer, and water were prepared in triplicate and incubated at 37°C for 15 minutes. After incubation, the triplicate reactions were separated into the following 3 groups: negative control, EndoH (New England BioLabs, P0702L), and PNGase F (New England BioLabs, P0704L). The EndoH samples received 4 μL 10× G3 reaction buffer, 12 μL water, and 4 μL EndoH (New England BioLabs, P0702L). In the negative control reactions, the EndoH was replaced with water. PNGase F reactions received 4 μL of 10× G2 reaction buffer, 4 μL 10% NP-40, 9 μL water, and 3 μL PNGase F. All reactions were placed at 37°C for 3 hours. Reactions were terminated with 15 μL 4× NuPAGE LDS sample buffer (Invitrogen, 2152677) containing DTT (MilliporeSigma, D0632-10G) before Western blot analysis or storage at −80°C.

### Western blot

To visualize NPC1, 40 μg protein was loaded per well and then separated on a NuPAGE 4%–12% gradient Bis-Tris gel (Invitrogen, WG1401BX10). The gel was transferred to Immobilon-P 0.45 μm PVDF (Merck Millipore, IPVH00010) membrane, placed in a blocking solution of 5% nonfat milk for 1 hour, and then placed in a solution of block plus appropriate primary antibody overnight. The next day, membranes were rinsed in approximately 15 mL TBST 3 times before placement in appropriate secondary antibody for 1 hour. After another 3 rinses in TBST, immunoreactivity was detected with SuperSignal West Pico PLUS Chemiluminescent Substrate (Thermo Scientific, 34577) via an iBright FL1500 imaging system (Invitrogen, A44241). Quantification of bands within the linear exposure range was performed using ImageJ (NIH), and band intensity was normalized to the indicated loading control. If brightness and contrast were modified, adjustments were performed equally to the entire image and controls after quantification.

### Fluorescence staining

#### Filipin staining.

Fibroblasts and Hap1 cells were stained with filipin and lectin wheat germ agglutinin (WGA) to outline plasma membrane as described previously (30). iNeurons were washed twice with DPBS+/+, fixed with 4% PFA for 20 minutes at room temperature, and then washed twice with DPBS+/+. Cells were permeabilized with 0.1% Triton X-100 for 3 minutes, blocked in 10% normal goat serum plus 1% BSA for 40 minutes, and then incubated with primary antibody solution diluted in block overnight at 4°C. The following day, cells were washed twice in DPBS+/+ and stained for 2 hours with filipin solution (1 mL FBS + 8 mL DPBS + filipin [1 μg filipin + 30μL DMSO]) and secondary antibody. After washing twice in DPBS+/+, cells were mounted with ProLong Gold (Thermo Fisher, P36930) and imaged.

### Microscopy

Following staining with filipin or anti-GM2 antibody, images were focused with WGA or TuJ1, and tiled images were captured and analyzed as previously described (30).

### Lipid analysis by LC-MS

#### Materials.

Methanol (LC-MS grade), chloroform (LC-MS grade), ammonium acetate (reagent grade), ammonium formate (reagent grade), formic acid (reagent grade), acetonitrile (LC-MS grade), phenylmethylsulfonyl fluoride (PMSF), and protease inhibitor cocktail tablets (S8820) were from MilliporeSigma. Isopropanol (LC-MS grade) was from VWR international. Deuterated lipid standard mixture (SPLASH lipidomix mass spec standard, 330707) was from Avanti Polar Lipids. BCA protein assay was from Pierce (23225, Thermo Scientific). Oasis HLB solid-phase extraction cartridges were from Waters (WAT094225).

#### Lipid extraction for LC-MS.

iNeuron cell pellets were homogenized using probe sonication in 1× PBS buffer containing protease inhibitors and phosphatase inhibitors [1 mM NaF, 1 mM β-Glycerophosphate, 1 mM PMSF, 1 mM Na_3_VO_3_, 2.5 mM Na_4_(PO_4_)_2_]. Cell lysates were cooled on ice in between sonications. Protein concentration was measured using the Pierce BCA Protein Assay. 60 μg protein equivalent from each cell lysate was taken, and 5 μL SPLASH lipidomix mass spec standard was added. The volume of each 60 μg protein lysate was adjusted to 150 μL using 1× PBS, and 600 μL of a chloroform/methanol (1:2, v/v) mixture was added. Samples were sonicated in a bath sonicator 3 minutes, and the sonication was repeated 2 more times. At the end of the sonication samples were centrifuged for 1 minute at 20,000 rcf to pellet the protein, supernatants were transferred into new tubes, and 100 μL 1× PBS was added. Samples were centrifuged for 2 minutes at 18,407*g* to induce the phase separation. The lower, organic phase was dried and resuspended in 40 μL of chloroform/methanol (1:3, v/v) and analyzed in LC-MS for the cholesterol quantification. The upper aqueous phase was taken for desalting using oasis HLB solid-phase extraction cartridges. The HLB cartridges were conditioned by passing 1 mL methanol and equilibrated with 1 mL water. The upper aqueous phase was loaded onto the cartridge and washed by passing 1 mL 5% methanol. Gangliosides were eluted using 2 mL methanol and dried under vacuum. Dried samples were resuspended in 40 μL of 90% methanol and transferred into autosampler vials for LC-MS analysis.

#### LC-MS analysis of cholesterol.

Lipid separation was performed using an Agilent 1260 UHPLC system outfitted with an Agilent Eclipse plus C18 column (3 × 50 mm, 1.8 μm). The flow rate was set to 400 μL/min, and the column was maintained at 50°C. Liquid chromatography mobile phases were composed of solvent (A), which contained water with 5 mM ammonium formate and 0.1% (v/v) formic acid, and solvent (B), which contained 50:50 (v/v) isopropanol/methanol with 5 mM ammonium formate and 0.1% (v/v) formic acid. From each sample, 2 μL was injected into the chromatographic system and analyzed using an Agilent 6550 quadrupole time-of-flight mass spectrometer in positive ion mode *m/z* range of 200–1,700.

#### LC-MS analysis of gangliosides.

Lipid separation was performed using an Agilent 1260 UHPLC system outfitted with an Agilent Poroshell 120 EC-C18 column (2.1 × 100 mm, 2.7 μm). The flow rate was set to 300 μL/min, and the column was maintained at 50°C. Liquid chromatography mobile phases were composed of solvent (A), which contained water with 20 mM ammonium acetate, and solvent (B), which contained 20:80 (v/v) isopropanol/methanol with 20 mM ammonium acetate. From each sample, 3 μL was injected into the chromatographic system and analyzed using an Agilent 6550 quadrupole time-of-flight mass spectrometer in negative ion mode *m/z* range of 200–1,700.

### Mice

Previously described (34) *Npc1*-I1061T mice were backcrossed to C57BL/6 (≥10 generations).

### Data and materials availability

The data and materials supporting the findings of this study are available from the corresponding authors upon reasonable request.

### Statistics

GraphPad Prism (v9.3.1) was used to analyze statistical differences. A single comparison was analyzed by a 2-tailed *t* test and multiple comparisons were analyzed by a 1-way ANOVA with Tukey’s post hoc test. Groups with multiple variables were analyzed by a 2-way ANOVA with Bonferroni’s post hoc test. Normal distribution of data was assumed. *P* values of less than or equal to 0.05 were considered significant.

### Study approval

All procedures involving mice were approved by the University of Michigan Committee on Use and Care of Animals (PRO00010017) and conducted in accordance with institutional and federal guidelines. Patient fibroblasts with the R934L/R1007X *Npc1* mutation were collected under REB no. 1000052878 (The Hospital for Sick Children, Toronto, Ontario, Canada). For additional primary patient I1061T/I1061T fibroblasts, fibroblasts were obtained via skin biopsy from individuals enrolled in the Natural History/Observational study (NCT00344331) at the NIH. This study was approved by the National Institute of Child Health and Human Development and NIH Institutional Review Boards. Written informed consent was obtained from guardians or participants.

## Author contributions

MLS, EAI, FDP, SMC, MDU, and APL conceptualized the study. MLS, MDU, SMC, and APL provided methodology. MLS, EQK, KJS, RDA, SE, EAI, NYF, KCP, and MDU provided investigation. MLS, SMC, APL, and FDP acquired funding. MLS, EAI, FDP, SMC, MDU, and APL administered the project. MLS, EAI, FDP, SMC, and APL supervised the study. MLS, EQK, KJS, RDA, and APL wrote the original draft of the manuscript. MLS, EQK, KJS, RDA, SE, EAI, NYF, FDP, KCP, SMC, MDU, and APL reviewed and edited the manuscript.

## Supplementary Material

Supplemental data

## Version 1 10/27/2022

In-Press Preview

## Version 2 12/08/2022

Electronic publication
