# Supplementary material for: Species-specific differences in NPC1 protein trafficking govern therapeutic response in Niemann-Pick type C disease
Source: JCI Insight. 2022 Dec 8;7(23):e160308. doi: 10.1172/jci.insight.160308 (PMC9746915; doi:10.1172/jci.insight.160308)
Supplement: Supplemental data [file jciinsight-7-160308-s110.pdf]

Supplementary Methods and Data

Species-specific differences in NPC1 protein trafficking govern therapeutic response in Niemann-Pick type C disease

Authors: Mark L. Schultz[1,*], Kylie J. Schache[1], Ruth D. Azaria[1], Esmée Q. Kuiper[1], Steven Erwood[2,3], Evgueni A. Ivakine[2,4], Nicole Y. Farhat[5], Forbes D. Porter[5], Koralege C. Pathmasiri[6], Stephanie M. Cologna[6], Michael D. Uhler[7,8], Andrew P. Lieberman[1,*]

Affiliations:
[1]*Department of Pathology, University of Michigan Medical School, Ann Arbor, Michigan, USA.*

[2]*Program in Genetics and Genome Biology, The Hospital for Sick Children Research Institute, Toronto, Ontario, Canada.*

[3]*Department of Molecular Genetics, University of Toronto, Toronto, Ontario, Canada.*

[4]*Department of Physiology, University of Toronto, Toronto, Ontario, Canada.*

[5]*Division of Translational Medicine, Eunice Kennedy Shriver National Institute of Child Health and Human Development, National Institutes of Health, DHHS, Bethesda, Maryland, USA.*

[6]*Department of Chemistry, University of Illinois Chicago, Illinois, USA.*

[7]*Michigan Neuroscience Institute, University of Michigan Medical School, Ann Arbor, Michigan, USA.*

[8]*Department of Biological Chemistry, University of Michigan Medical School, Ann Arbor, Michigan, USA.*

[*]*Corresponding authors. Email: scmark@med.umich.edu; liebermn@med.umich.edu*

Supplementary methods

I1061T mice: I1061T mice were genotyped as previously described (1). For liver collection, 6-week-old mice were anesthetized, perfused with saline, and livers were flash frozen in liquid nitrogen. Livers were then homogenized, sonicated in RIPA, and non-dissociated tissue was removed by centrifugation at 3000x*g* 4°C for 5 minutes. Protein concentration of the supernatant was calculated using DC™-protein assay (Bio-Rad) and normalized. 50 µg of lysate was added to each reaction and EndoH was performed as in the main text.

RT-qPCR: TRIzol® (Thermo Fisher 15596018) was used to collect RNA. RNA was converted to cDNA using the High Capacity Reverse Transcription kit (Applied Biosystems 4368814). Quantitative real-time PCR was conducted in technical replicates using 10 ng cDNA, TaqMan™ probes (Thermo Fisher) for 18s (4310893), TBP (Hs00427620, Mm01277042), NPC1 (Hs00264835, Mm00435300), Syn1 (Hs01018066), Map2 (Hs00258900), and MapT (Hs00902194). RT-qPCR was performed using ABI 7900HT Sequence Detection System and relative expression was calculated by the 2^ (−delta delta Ct) method using SDS software.

Pluripotency staining: The Pluripotent Stem Cell 4-Marker Immunocytochemistry Kit (ThermoFisher A24881) was used following the manufacturer's instructions.

iNeuron differentiation staining: Cells were washed 3X with DPBS+/+ and fixed with 4% PFA for 20 minutes. After 3 washes, cells were permeabilized using 0.1% trition for 3 minutes then blocked (10% NGS, 1% BSA). Cells were incubated overnight at 4ºC with MAP2 antibody (Cell Signaling 4542, 1:50) diluted in block solution. The following day cells were washed 3 times in DPBS+/+ then labeled with secondary antibody at 1:500 for 1 hour at room temperature. Cells were washed 3 times in DPBS+/+ and mounted with VectaShield containing DAPI.

Karyotyping: Chromosomes were harvested and analyzed using a GTW banding methods. Of 20 metaphase cells analyzed, all were characteristic of a chromosomally normal male karyotype. Three of these metaphase spreads were digitally processed to produce a karyotype/karyogram to perform a detailed analysis for variant counts and structural aberrations.

Cell Viability: iNeurons were treated with vehicle or dantrolene for 5 days and cell viability was determined using the CyQuant Xtt Cell Viability Assay (Thermo Fisher X12223) following the manufacturer's instructions.

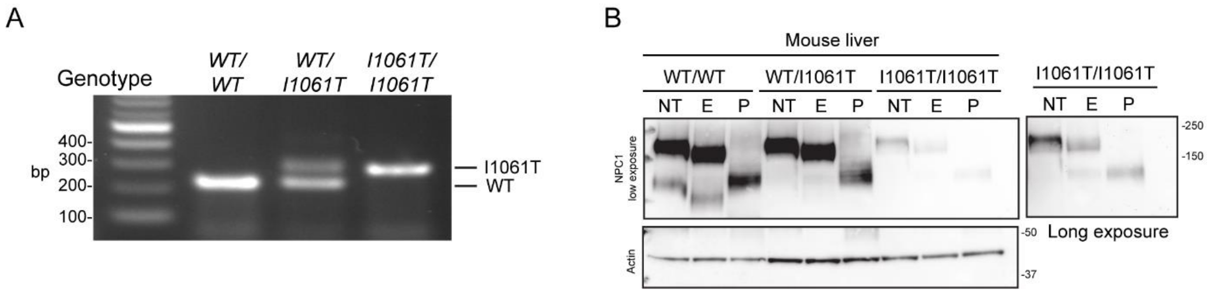

Supplementary Figure 1. Confirmation of mouse I1061T-NPC1 trafficking. (A) I1061T mouse genotyping**.** (**B)** Liver lysates from WT (WT/WT), I1061T heterozygous (WT/I1061T), or I1061T homozygous (I1061T/I1061T) were incubated with no treatment (NT), or digested with EndoH (E), or PNGase F (P), then subject to Western blot.

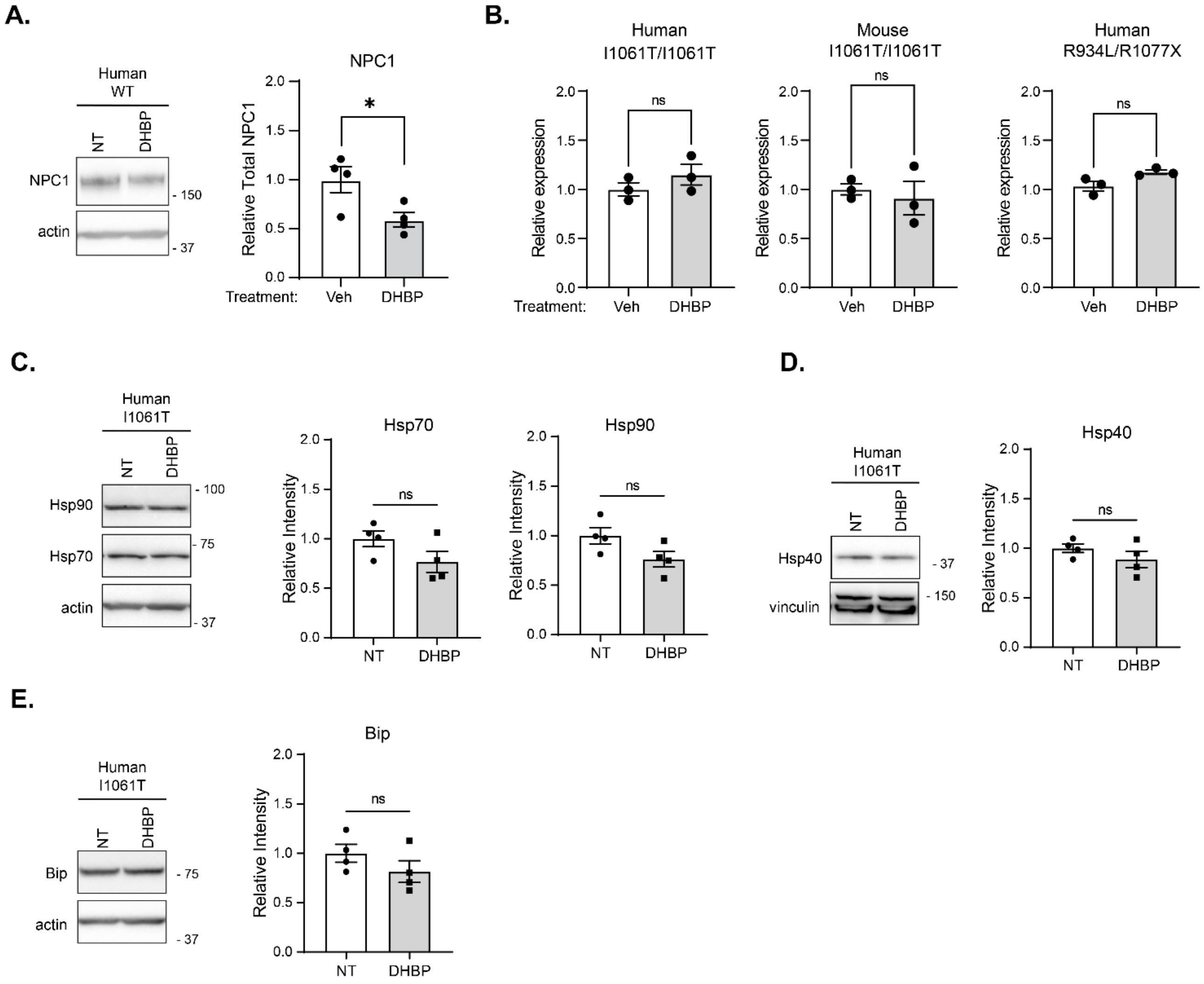

Supplementary Figure 2. DHBP does not alter *NPC1* gene expression or chaperones. Primary human and mouse I1061T/I1061T or human R934L/R1077X fibroblasts were treated with 5 μM DHBP (daily) or with vehicle (PBS) for five days, and NPC1 mRNA expression (normalized to TBP) was analyzed by qPCR. Data are mean ± s.e.m. from **A.** 4, **B.** 3, **C.** 4, **D.** 4, and **E.** 4 independent experiments. n.s., not significant, $^*P \leq 0.05$ by two-tailed t test **A.** t= 2.7, df=6; **B.** t=1.2, 0.5, 2.6; DF=4; **C.** t= 1.6, 2.1, df=6; **D.** t=1.2, df= 6; **E**. t=1.3, df=6.

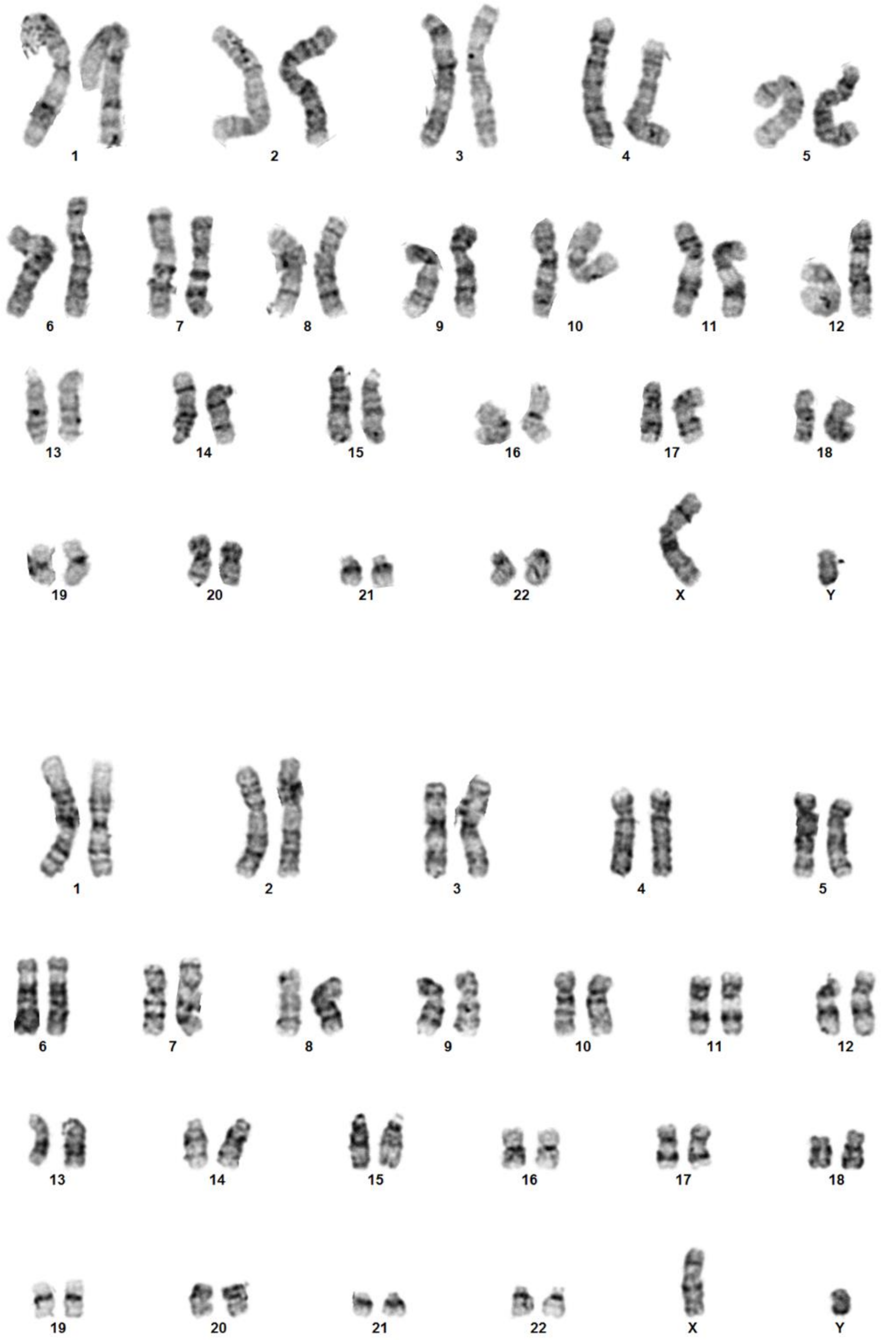

I1061T

R934L

Supplementary Figure 3. CRISPR edited iPSCs have a normal karyotype. Karyotypes of I1061T and R934L clone 1 iPSCs were analyzed by the Cytogenetics core at Washington University Saint Louis. The WT iPSC karyotype has been previously described (2).

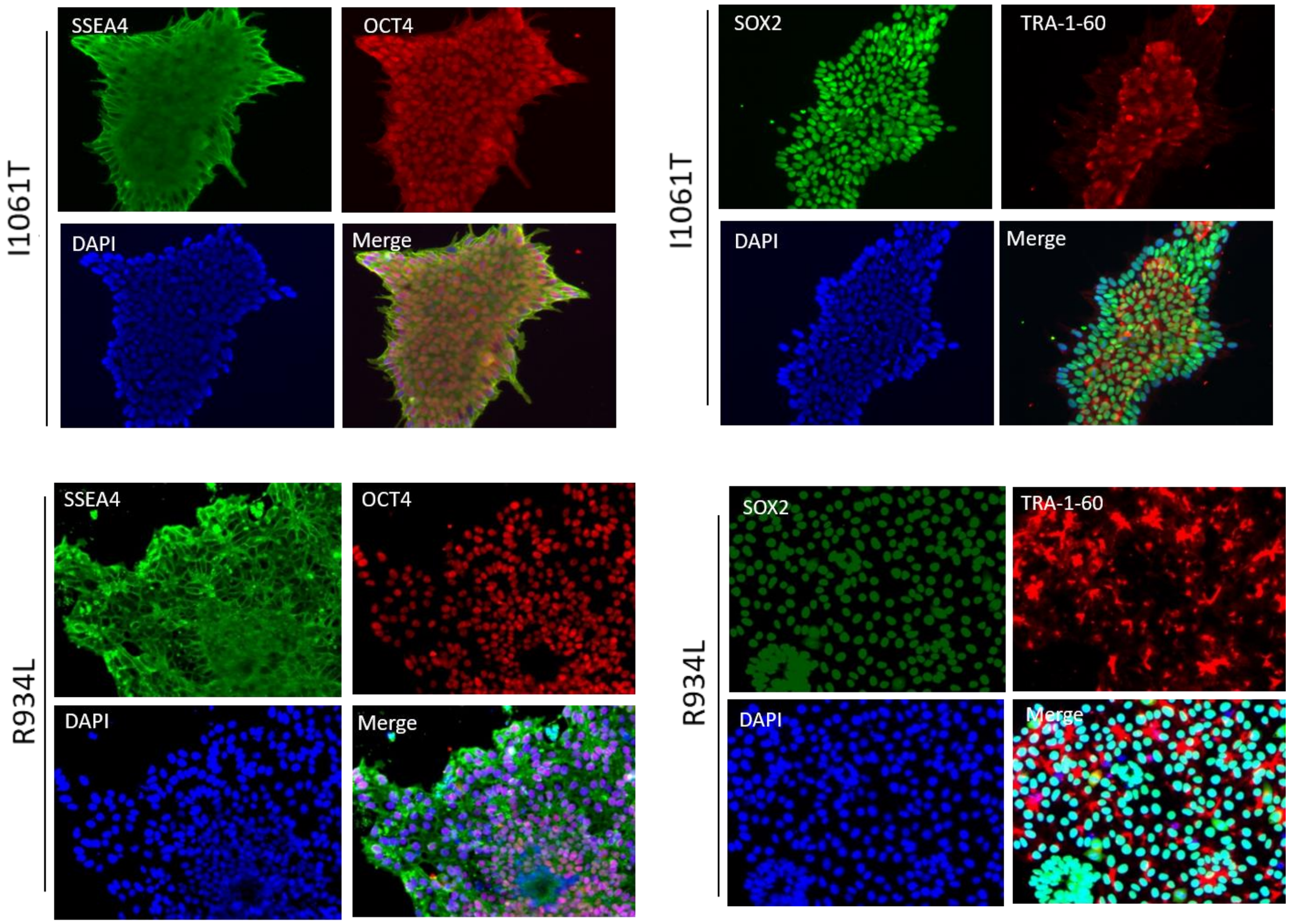

Supplementary Figure 4. Pluripotency analysis of iPSC lines. I1061T or R934L iPSCs were stained with the pluripotency markers SSEA4 (left green), OCT4 (left, red), SOX2 (right green), TRA-1-60 (right red), or DNA with DAPI (blue). Positive immunoreactivity for SSEA4, OCT4, SOX2 and TRA-1-60 indicates that both lines are pluripotent. WT iPSC pluripotency has been previously described (2).

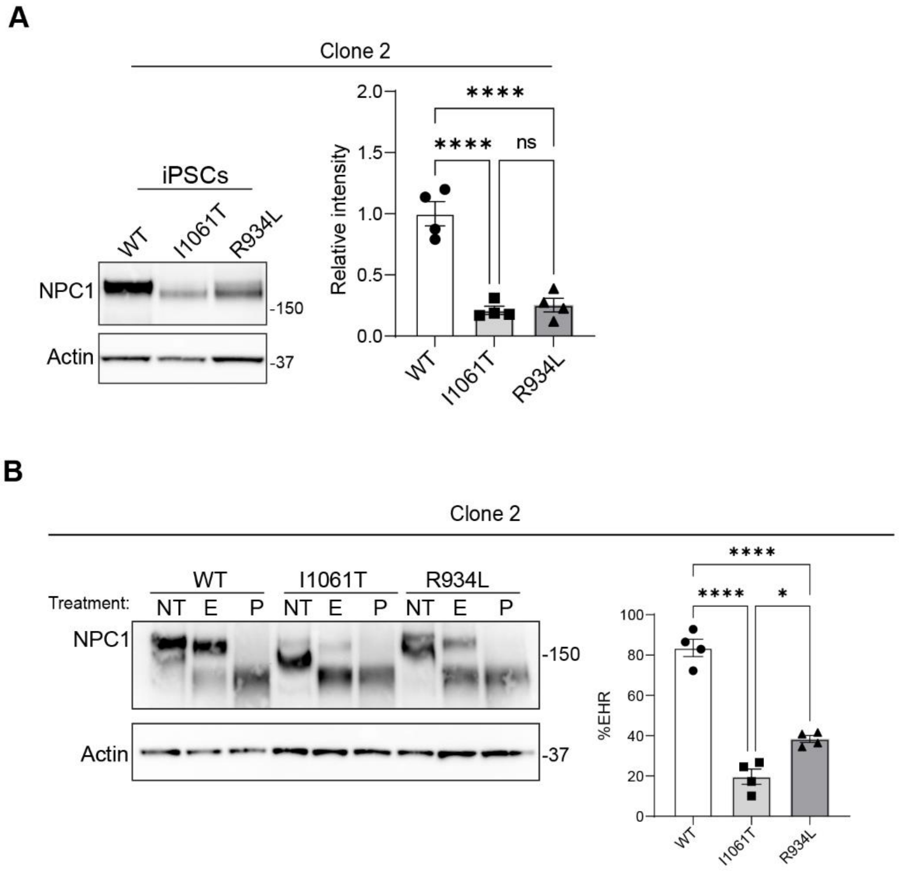

Supplementary Figure 5. Characterization of NPC1 trafficking in multiple iPSC clones. Lysates from an additional independent clone for each mutation (I1061T or R934L) were analyzed for **A**. total NPC1 (quantified at right), or **B**. incubated with no treatment (NT) or digested with EndoH (E) or PNGase F (P), then subjected to western blot (quantified at right). Data are mean ± s.e.m. from 4 independent experiments. n.s., not significant, *$P \leq 0.05$, ***$P \leq 0.001$, ****$P \leq 0.0001$ by ANOVA with Tukey's posthoc; **A.** F=111.6, 42.05; DF=2. **B.** F=90.45, 152.5; DF=2.

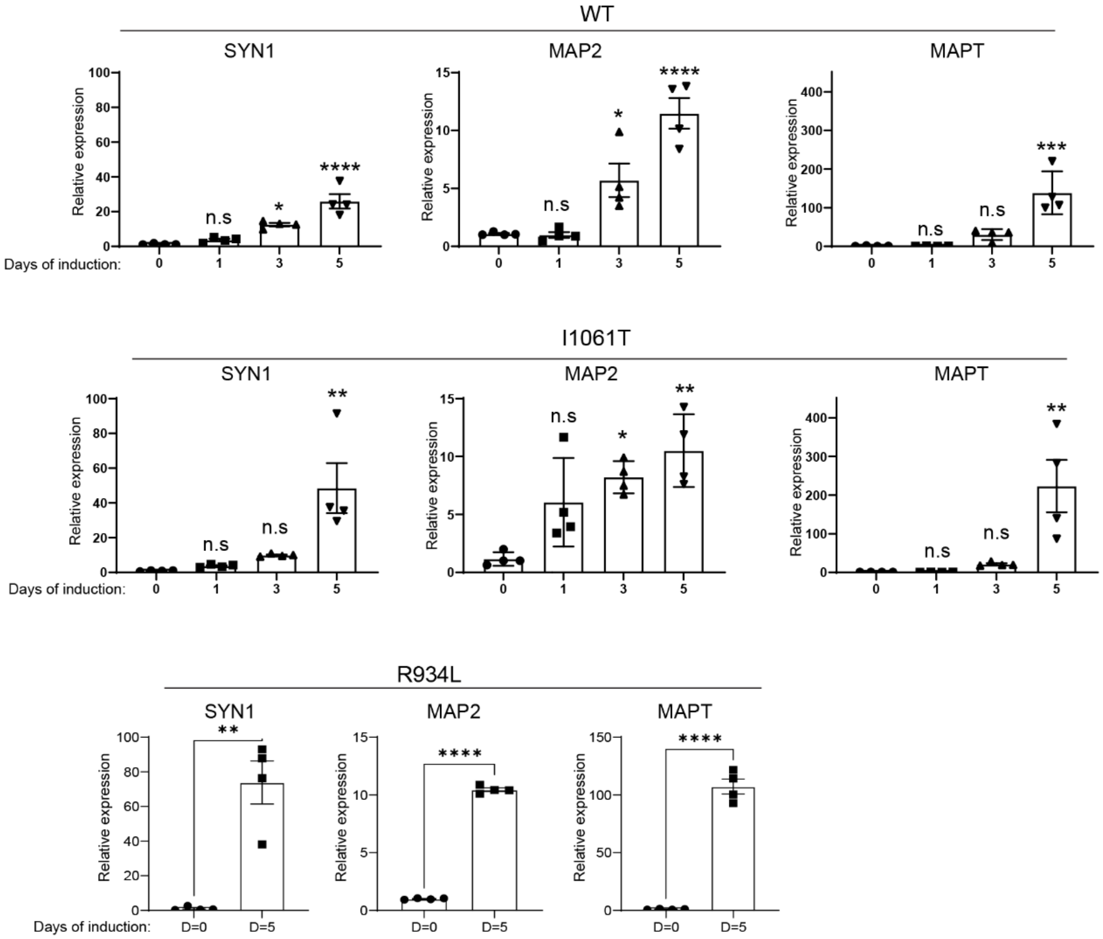

Supplementary Figure 6. Characterization of iPSC iNeuron clones. iNeuron differentiation was confirmed by qPCR of WT, I1061T, and R934L iNeuron iPSCs at day 0, 1, 3, and 5 post differentiation using probes for SYN1, MAP2, and MAPT. Data are mean ± s.e.m. from 4 independent experiments. n.s., not significant, **P* ≤ 0.05, ****P* ≤ 0.001, *****P* ≤ 0.0001 by WT, I1061T=ANOVA with Tukey's posthoc or R934L t-test. WT, I1061T: F=26.7, 25.2, 21, 9.4, 9.5, 10.2, DF=3, R934L t=5.9, 58.1, 16.32, DF=6.

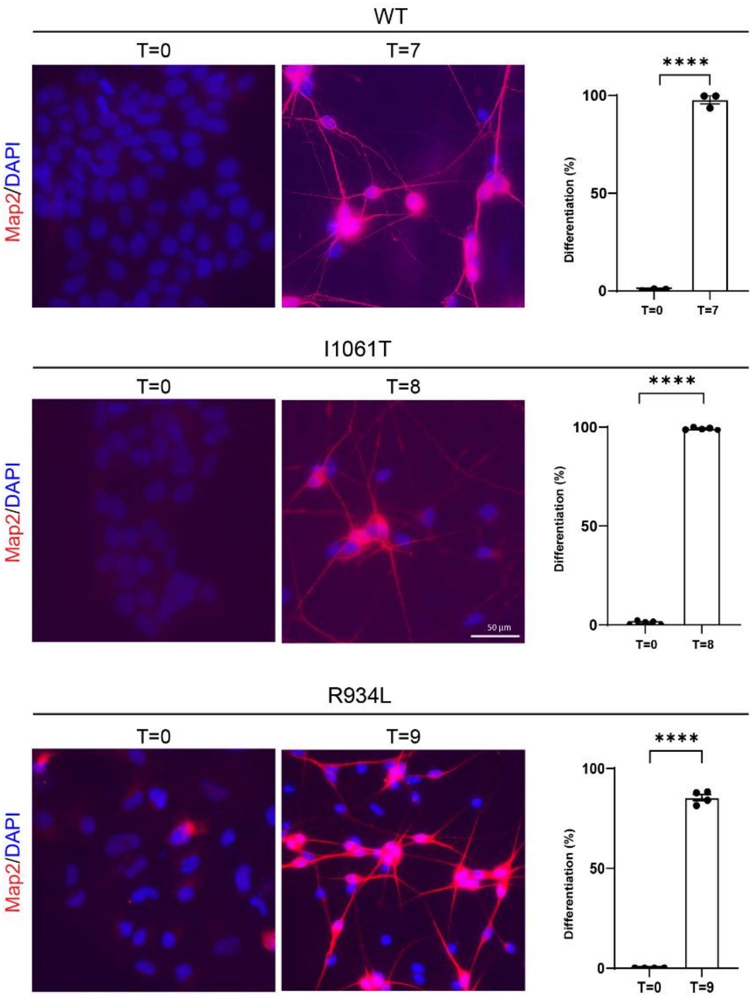

Supplementary Figure 7. Imaging characterization of iPSC iNeuron clones. WT, I1061T, and R934L iPSC iNeurons were untreated (T=0) or treated with doxycycline for 7-9 days and stained with DAPI (blue) or MAP2 (red). Percentage of MAP2 positive somas with projections quantified at right. Data are mean ± s.e.m. from 3, 5, or 4 independent experiments, ****$P \leq 0.0001$ by t-test. (WT) t=44.93, df=4, (I1061T) t=178.1, df=8, (R934L) t=53.0, df=6.

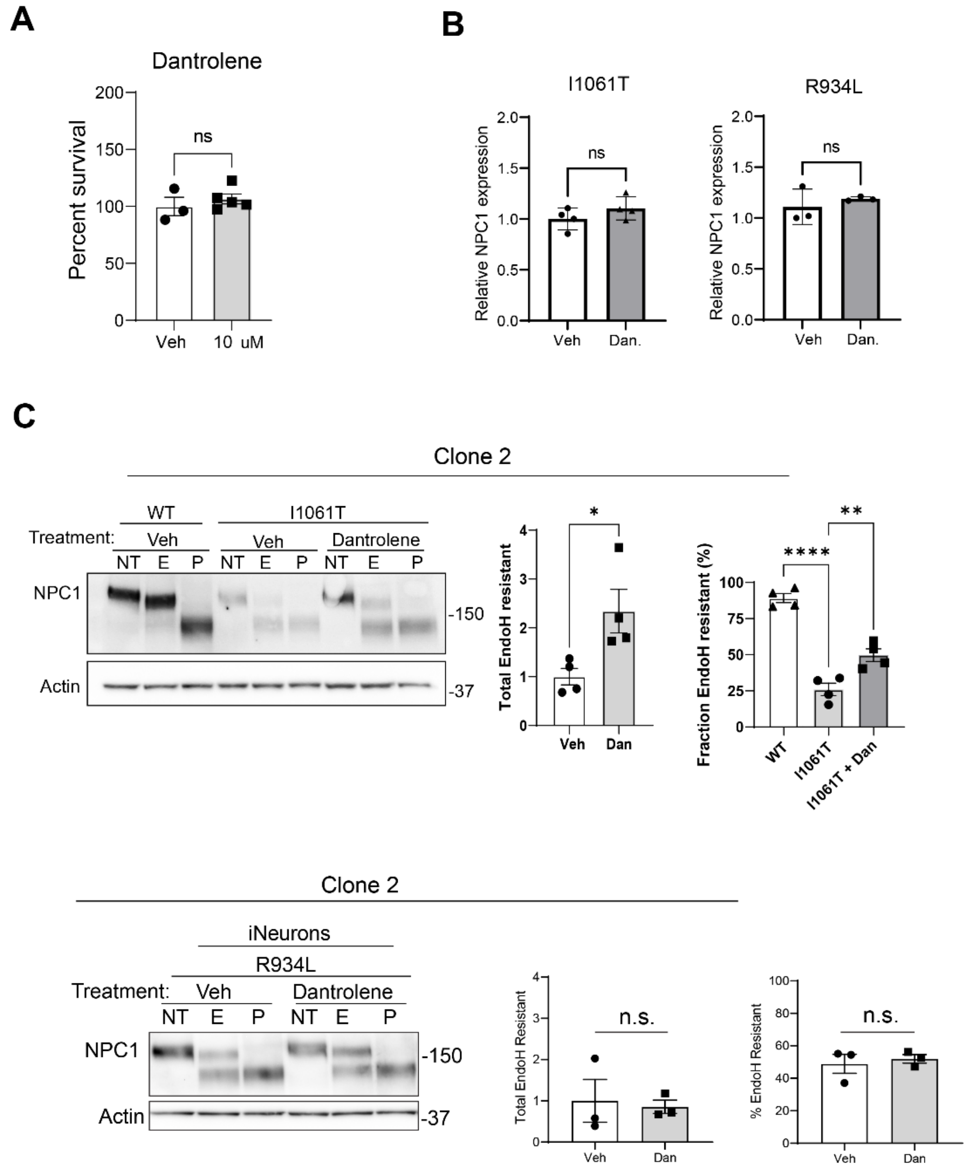

Supplementary Figure 8. RyR antagonist treatment of iPSC iNeurons. A. I1061T-NPC1 iNeurons were treated with vehicle (Veh) or dantrolene (10 uM) for 5 days and cell viability was measured using the CyQUANT XTT Assay. **B.** NPC1 gene expression (qPCR) of I1061T or R934L with Veh or dantrolene (Dan) treatment for 5 days. **C.** Clone 2 of WT, I1061T, or R934L iNeurons were treated with Veh or Dan for 5 days and lysates were collected and analyzed by EndoH assay (quantified at right). Data are mean ± s.e.m. from n=3-5 independent experiments, $*P \leq 0.05$, $**P \leq 0.01$, $***P \leq 0.001$, $****P \leq 0.0001$ by t-test or ANOVA with Tukey's posthoc. **A.** t=0.8, df=6. **B.** t=1.3, df=6. t=0.8, df=4. **C.** t=2.8, df=6, F=63.9, df=2; t=0.2, 0.4, df=4.

STR	WT (Parental)	I1061T	R934L
AMELX	X, Y	X, Y	X, Y
PentaE	7, 17	7, 17	7, 17
D21S11	29	29	29
D19S433	16	16	16
D13S317	8, 9	8, 9	8, 9
TPOX	10, 11	10, 11	10, 11
D7S820	11, 12	11, 12	11, 12
D5S818	12	12	12
CSF1PO	10, 12	10, 12	10, 12
D16S539	9, 13	9, 13	9, 13
D3S1358	14, 16	14, 16	14, 16
TH01	7, 8	7, 8	7, 8
D8S1179	9, 11	9, 11	9, 11
VWA	16, 18	16, 18	16, 18
FGA	22, 23	22, 23	22, 23
D2S1338	16, 20	16, 20	16, 20
D18S51_#1	17, 19	17, 19	17, 19

Supplementary Table 1. Short tandem repeat analysis of iPSC lines. Short tandem repeat (STR) analysis of 16 loci was performed by Genome Engineering and iPSC center (GEiC) at Washington University in St. Louis.

Supplementary References:

1. Praggastis M, Tortelli B, Zhang J, Fujiwara H, Sidhu R, Chacko A, et al. A murine Niemann-Pick C1 I1061T knock-in model recapitulates the pathological features of the most prevalent human disease allele. *J Neurosci.* 2015;35(21):8091-106.
2. Chen YH, and Pruett-Miller SM. Improving single-cell cloning workflow for gene editing in human pluripotent stem cells. *Stem Cell Res.* 2018;31:186-92.
